# Learning style preference and the academic achievements of medical students in an integrated curriculum

**DOI:** 10.25122/jml-2023-0366

**Published:** 2023-12

**Authors:** Adel Mohamed Aboregela

**Affiliations:** Department of Basic Medical Sciences, College of Medicine, University of Bisha, Bisha, Saudi Arabia

**Keywords:** PBL, undergraduates, GPA, VARK

## Abstract

Understanding how individuals learn best, known as learning style, is integral to optimizing educational outcomes. This analytical study was conducted among students in their fourth year who finalized their problem-based activities at the College of Medicine, University of Bisha, Saudi Arabia. The visual, aural, read/write, and kinesthetic (VARK) model was adopted to assess individual differences in learning preferences and their correlation with academic achievement in the problem-based learning (PBL)-dependent curriculum. The online self-administered survey was completed by 64 students with a response rate of 79%. Of these, 63.5% were men and 36.5% were women, with a mean age of 21.9 years and a grade point average (GPA) of 3.83. Analysis of learning style distribution revealed that 34.9% preferred visual, 54% preferred auditory, 17.5% preferred read/write, and 90.5% preferred kinesthetic styles. Also, combined learning modalities revealed that 14.3% preferred unimodal, 74.6% bimodal, and 11.1% trimodal approaches. The most frequent unimodal approach was kinesthetic, while auditory/kinesthetic and visual/auditory/kinesthetic were the predominant bimodal and trimodal preferences. No significant differences in GPA were found among students with different selective learning styles or combined learning modalities, as determined by one-way ANOVA and chi-square tests. Spearman's rho correlation revealed a positive correlation between the learning modality and the auditory style (*P* < 0.001). Also, a negative correlation was identified between reading/writing versus kinesthetic and auditory versus visual learning styles (*P* = 0.001). However, no significant correlations were identified between grades or GPA and specific learning styles. It can be concluded that the integrated PBL-dependent curriculum adopted at the College of Medicine, University of Bisha, is a suitable teaching modality satisfying different learning styles, but continuous monitoring is crucial.

## INTRODUCTION

To facilitate better knowledge dissemination, education has shifted from the conventional teacher-centered approach to an integrated, student-centered model that fosters support and encourages learners [1]. Measuring learning achievements is one of the most critical indicators to evaluate the success of the learning process [2]. Learning outcomes can be significantly influenced by a variety of internal factors, as well as external environmental influences. Learning style preference is a major internal factor affecting learning outcomes [3]. The learning style is the unique physiological method by which a person can perceive, realize, approach, evaluate, and retain information [4]. Identifying students' learning styles has recently been considered a cornerstone in the learning process to improve performance, enhance engagement, minimize learning time, and boost outcomes [5].

Several authors have proposed and validated variable scales to evaluate students' learning approaches and perceptions. For instance, Riechman and Grasha scales categorized learning styles into six types: avoidant, competitive, participant, collaborative, dependent, and independent [6]. Gregorc divided the population into four sections according to their mind's ability to perceive and process information: concrete random, concrete sequential, abstract random, and abstract sequential [7]. Also, Fleming described the visual (V), aural (A), read/write (R), and kinesthetic (K) models of learning [8]. The Dunn and Dunn learning style model identified emotional, environmental, physiological, sociological, and psychological influences affecting learning [9]. In addition, Kolb outlined a four-step learning process: concrete learning, reflective observation, abstract conceptualization, and active experimentation [10], while McCarthy's model focused on perceiving and processing information through four different learner appeals: why, what, how, and what if [11]. Moreover, the index of learning styles assessed learning preferences based on four dimensions: active/reflective, sensing/intuitive, visual/verbal, and sequential/global [12]. Reid’s perceptual learning styles were built on using one or more senses (auditory, visual, kinesthetic, tactile) to understand, organize, and retain knowledge [13]. One of the most widely used instruments, described by Fleming and Baume (VARK), categorizes learning preferences into four perception styles. For example, visual learners learn by observing images, figures, and videos, while aural learners thrive by listening to lectures. Reading-writing learners prefer to absorb information through reading texts and taking notes, whereas kinesthetic learners learn best by touching and manipulating objects during learning [14].

Several studies reported different factors with bimodal relationships with the preferred learning style. The type of curriculum is considered one such debated factor. Controversial results were reported regarding the preference for problem-based learning (PBL) curricula as a multimodal approach over lecture-based curricula in accommodating a broader range of learning styles and dealing with a deeper approach to learning [15-17]. Also, various studies elaborated on the great debate regarding academic achievement as an influencing factor in learning styles [1, 18, 19]. This discrepancy has raised the need for further investigations to assess the multifactorial relationship between the student's learning style and academic achievement, considering the type of curriculum. These investigations could add to the body of literature and be valuable in aligning teaching modalities with learners' needs to achieve better outcomes. The College of Medicine, University of Bisha, Saudi Arabia, adopted an integrated, student-centered, PBL-dependent curriculum with a multi-strategy approach, which requires continuous evaluation and refinement [20]. To improve the learning experience, this study was designed to assess individual differences between learners according to the VARK model and to evaluate their associations with sociodemographic characteristics and academic achievement by the end of the PBL-dependent phase of the curriculum.

## MATERIAL AND METHODS

A cross-sectional analytical study was conducted at the College of Medicine, University of Bisha, Saudi Arabia. The study population included all students registered in the second semester of the fourth year during the academic year 2022-2023 (81 students). The sample was comprehensive and homogenous in academic background and age. The first inclusion criteria required that students successfully completed their first semester without failure, postponement, or prohibition, and the second criterion was the willingness to participate in the study. The integrated curriculum at the College of Medicine, University of Bisha, Saudi Arabia, consists of two main phases. The first phase includes teaching activities such as PBL, team-based learning (TBL), and seminars, and the second phase includes case-based learning (CBL), TBL, and seminars. Fourth-year students were selected because they finalized the first phase of their integrated curriculum, including all the PBL activities requested.

A pre-validated, self-administered online questionnaire was developed using Google Forms and distributed to all students via WhatsApp. The questionnaire started with an introduction explaining the objectives of the study, consent, voluntary participation, anonymity, confidentiality of responses, and the option for participants to learn about their learning style post-analysis. The first section of the survey included primary sociodemographic characteristics, including age, gender, and Grade Point Average (GPA) obtained at the end of the last semester. The second section included the previously validated and published online VARK questionnaire version 8.0, which consists of 16 questions with four options representing various learning styles. Respondents could select multiple options based on their preferences in the described scenarios. The questions in the survey inquire about the preferred modality for dealing with specific situations, such as selecting a career or area of study, determining what is important for the individual, and identifying how they would learn most effectively from different instructional formats. For example, scenarios may involve deciding how to learn from a website featuring a video on creating a specialized graph or from someone verbally describing a task. Responses were analyzed to determine the percentage of each learning style for each participant, calculated by dividing the score of the learning style (ranging from 0 to 16) by the total score (ranging from 16 to 46). A learning style was considered predominant if its percentage exceeded 25% of the total score.

Data were retrieved from Google Forms, coded, and analyzed using Microsoft Excel and SPSS 21. Descriptive data were presented as frequencies. Comparisons between different sociodemographic characteristics were conducted using the Mann–Whitney U test for variables with two groups and ANOVA for more than two groups. The chi-square test and non-parametric Spearman's rho correlation assessed the relationship between different variables. Significance was considered when *P* < 0.05.

## RESULTS

**Figure 1:**
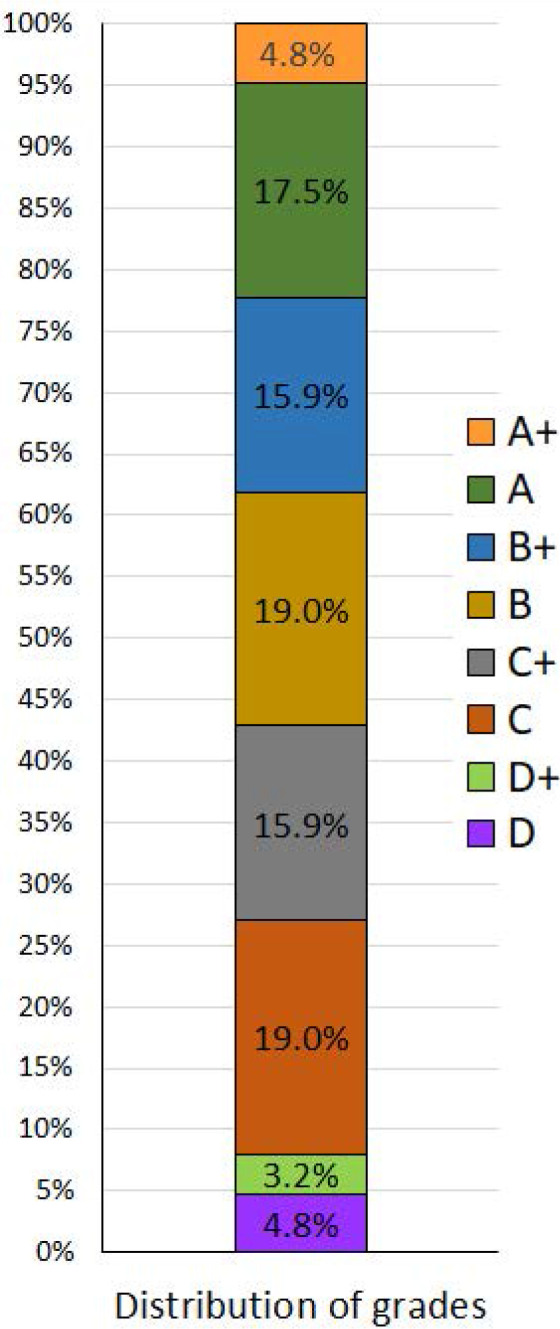
Graphical distribution of grades achieved by students at the end of the last semester Values are presented as percentages

The survey was distributed to a total of 81 students, resulting in a response rate of 78% (63 respondents). All participants were enrolled in level eight (second semester of year four) for the academic year 2022/2023 without any postponements or prohibitions. Among the respondents, 63.5% were male participants, and 36.5% were female participants. The mean age of students was 21.9 ± 0.928. Their GPA ranged from 2.50 to 4.91, with a mean ± SD of 3.83 ± 0.634. Grades B and C had the highest frequency among respondents (19% each), followed by grade A (17.5%) and grades B+ and C+ (15.9% each). It was interesting to find that the highest grade (A+) and the lowest grade (D) had the same frequency (4.8%). The frequency distribution of grades achieved by respondents is presented in Figure 1.

**Figure 2:**
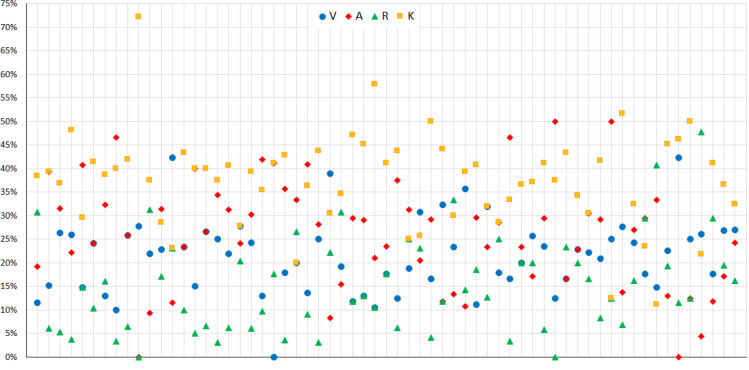
Student scores for selective learning styles based on the VARK questionnaire. Scores are presented as percentages on the vertical axis and students on the horizontal axis. V, visual; A, aural; R, read/write; and K, kinesthetic.

Selective learning style preferences were reported as follows: 34.9% visual, 54% auditory, 17.5% reading/writing, and 90.5% kinesthetic. Individual scores for each selective learning style were calculated based on preferences (Figure 2). No significant differences were observed between students with different grades or genders regarding their scores in selective learning styles. Additionally, there were no significant differences in GPA among students with different selective learning styles (e.g., visual versus non-visual). However, there was a significant difference in the mean age between auditory versus non-auditory and kinesthetic versus non-kinesthetic styles (*P* = 0.048 and 0.002, respectively). Furthermore, a significant gender-dependent GPA difference was obtained using the Mann–Whitney U test (*P* < 0.001). The means and standard deviations of variables with significant differences as determined by the Mann–Whitney U test are presented in Table 1.

**Table 1:** Comparison of GPA (Mean ± SD) across gender and age across learning styles with significant differences identified by Mann–Whitney U Test

**GPA**	**Male students** ***n* = 40 (63.5%)**	**Female students** ***n* = 23 (36.5%)**	**P value**
	3.57 ± 0.552	4.28 ± 0.503	< 0.001
**Age**	**Kinesthetic** ***n* = 57 (90.5%)**	**Non-kinesthetic** ***n* = 6 (9.5%)**	0.002
	21.79 ± 0.881	23.00 ± 0.635	
	**Auditory** ***n* = 34 (54%)**	**Non-auditory** ***n* = 29 (46%)**	0.048
	22.12 ± 0.978	21.66 ± 0.814	

GPA, Grade Point Average

**Figure 3:**
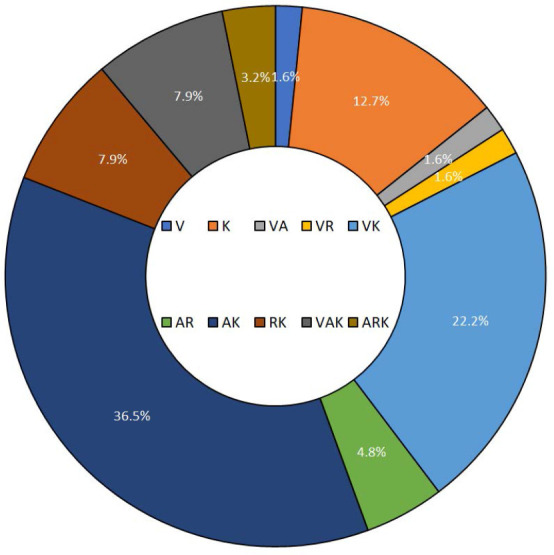
Distribution of combined learning styles among students. Values are presented as percentages. V, visual; A, aural; R, read/write; and K, kinesthetic.

Students had various learning styles, with 14.3% preferring a unimodal approach (single dominant learning style), 74.6% bimodal (two dominant learning styles), and 11.1% trimodal (three dominant learning styles). The unimodal kinesthetic learning style was the most prevalent among participants (12.5%), while the most frequent bimodal type was auditory/kinesthetic (35.94%), followed by visual/kinesthetic (21.88%). Also, visual/auditory/kinesthetic was the most frequent trimodal type (7.81%). The detailed distribution frequency of combined learning styles is shown in Figure 3. There were no significant differences between mixed learning modalities (unimodal, bimodal, and trimodal) and GPA, age, or gender as determined by the one-way ANOVA and post hoc Tukey test. In addition, there were no significant differences in GPA, age, and gender between the ten combined learning styles (V, K, VA, VR, VK, AR, AK, RK, VAK, and ARK). Additionally, no significant relationships were observed using the chi-square test. Further analysis was conducted using non-parametric Spearman's rho correlation to explore potential relationships between different parameters.

The results showed a positive moderate significant correlation between the learning modality and the auditory learning style (r = 0.513; *P* < 0.001). Also, there was a moderate negative correlation between reading/writing and kinesthetic learning styles (r = -0.421; *P* = 0.001). In addition, auditory and visual learning styles had a negative moderate correlation (r = -0.392; *P* = 0.001). No significant correlations were identified between learning styles and grade or GPA.

## DISCUSSION

Learners have different characteristics that influence how they acquire and process information during the learning process, called learning style preference [20]. While academic professors typically have expertise in the content of their specialized courses, they often lack sufficient knowledge about their learners' characteristics, which can hinder efforts to improve the learning process [22]. Continuous follow-up of the curriculum is crucial in medical schools, as adjusting the learning outcomes to the student's learning styles greatly impacts the learning process [23, 24]. Previous research has indicated a relationship between students' achievements and learning styles [21]. In line with this, the current study was conducted to assess the validity of the PBL-dependent curriculum implemented since 2014 at the College of Medicine, University of Bisha, Saudi Arabia, to accommodate different learning styles.

The first part of this study described the distribution of selective learning styles. Results revealed that 90.5% preferred kinesthetic learning, followed by 54% auditory, 34.9% visual, and 17.5% reading/writing. Interestingly, a previous study conducted in the same College of Medicine four years ago mentioned that the dominant learning style was aural, followed by kinesthetic [20]. This variance could be attributed to methodological differences, as the previous study employed probability sampling including all grades and only male students due to the admission policies at the time, unlike the present study. Additionally, some studies indicated a preference for kinesthetic learning followed by visual [25], while others suggest a preference for reading/writing over tactile learning [5]. Dental students in Saudi Arabia preferred aural rather than kinesthetic learning styles [4], and medical students preferred kinesthetic rather than aural [1, 26].

No significant difference in the distribution of different learning styles was observed when considering gender. However, previous studies reported gender-dependent significant differences in the preferred learning styles [1, 25]. Additionally, the current study found that 14.3% of students preferred unimodal learning, 74.6% preferred bimodal learning, and 11.1% preferred trimodal learning. Similar studies showed that more than 60% of students preferred multimodal learning, while adult learners preferred acquiring knowledge through different modalities [5, 27, 28]. The most frequent bimodal type in the current study sample was aural/kinesthetic (35.94%), followed by visual/kinesthetic (21.88%). The trimodal type with the highest frequency was visual/auditory/kinesthetic (7.81%). Combining aural/kinesthetic and visual/auditory/kinesthetic styles was the most prevalent [29]. Conversely, Rezigalla and Ahmed reported the dominant pattern as aural/ kinesthetic followed by visual/reading and visual/kinesthetic [20], while Nuzhat *et al*. mentioned that the bimodal aural/kinesthetic style was more dominant than visual/kinesthetic and the most prevalent trimodal style was visual/auditory/kinesthetic [30]. These differences might come from the diverse curricula adopted at each college and the various teaching methods used. It was reported that student’s tendency to shift from a certain learning style to another is affected by the content of the curriculum and the nature of the learning methods used [31]. Students with multimodal learning patterns benefit most from active learning strategies as they can tune themselves to different teaching styles [32].

The second part of this study used GPA as an indicator of academic achievement and correlated students' GPA after finishing the PBL phase of the curriculum with their learning style. The same correlation was conducted in previous studies and showed marked discrepancies among authors. In the current study, some significant correlations were made using the non-parametric Spearman's rho test, such as between the learning modality and the aural learning style, reading/writing, kinesthetic learning styles, and auditory versus visual learning styles. However, when using different statistical methods, no significant relationships or differences were identified between the learning styles and grades or GPA. This finding is consistent with a previous study conducted at the same college that revealed a non-significant correlation between student GPA and the preferred learning style [20].

On the other hand, Abouzeid *et al*. found a significant relationship between kinesthetic and aural learning styles and academic achievement in their study [1]. Similarly, Pellon *et al*. mentioned that students with the best grades usually adopt the kinesthetic learning styles [33]. Moreover, Akbar and Nasution identified a relationship between learning styles and the GPA of their students [2]. In addition, applying Kolb's learning styles revealed a significant correlation between different learning styles and academic achievement [34]. These findings might explain why teaching and learning strategies in their undergraduate curriculum encourage these learning styles and foster their academic achievement [1]. A major strength of the PBL-dependent curriculum adopted at the College of Medicine, University of Bisha, Saudi Arabia, is that it integrates attitude with knowledge and skills [35]. This constructivist learning environment is thought to accommodate all types of learning styles and modalities and positively affect the perception of students regarding their educational context [16].

One of the limitations of this study is the low sample size, as the college is a newly established institution with a limited number of student enrollments. Another limitation was that the study considered only student achievement in curriculum evaluation, irrespective of other psychometric parameters such as perception and engagement.

## CONCLUSION

The current study reinforced the importance of identifying students’ learning styles in medical schools, as these might affect their academic achievement. Kinesthetics is the most prevalent learning style in many medical educational bodies, but other modalities are also represented in reasonable proportions. This requires careful planning and implementation of the integrated curriculum to accommodate these diverse learning styles effectively. In addition, continuous monitoring of students is crucial as there is a tendency to change the learning style according to the applied curriculum. Finally, the study added compelling evidence to support the effectiveness of a PBL-dependent curriculum, which can accommodate and satisfy different learning modalities.

## References

[1] Abouzeid E, Fouad S, Wasfy NF, Alkhadragy R, Hefny M, Kamal D (2021). Influence of personality traits and learning styles on undergraduate medical students' academic achievement. Adv Med Educ Pract.

[2] Akbar RR, Nasution ES (2021). Correlation learning style with grade point average fourth year medical student. Open Access Maced J Med Sci.

[3] Sarabi-Asiabar A, Jafari M, Sadeghifar J, Tofighi S, Zaboli R, Peyman H (2014). The relationship between learning style preferences and gender, educational major and status in first year medical students: a survey study from Iran. Iran Red Crescent Med J.

[4] Nasir F, Gulfam F, Ayub A, Abdullah Z, Ghani S, Ullah H (2021). The preferred learning styles among the undergraduate dental students at Foundation University College of Dentistry, Islamabad. Ann PIMS-Shaheed Zulfiqar Ali Bhutto Med Univers.

[5] Ojeh N, Sobers-Grannum N, Gaur U, Udupa A, Majumder MAA (2017). Learning style preferences: a study of pre-clinical medical students in Barbados. J Adv Med Educ Prof.

[6] Riechmann SW, Grasha AF (1974). A rational approach to developing and assessing the construct validity of a student learning style scales instrument. J Psycho.

[7] Gregorc AF, Butler KA (1984). Learning is a matter of style. VocEd.

[8] Fleming ND, Mills C (1992). Helping students understand how they learn. Teach Prof.

[9] Dunn R, Griggs SA, Olson J, Beasley M, Gorman BS (1995). A meta-analytic validation of the Dunn and Dunn model of learning-style preferences. J Educ Res.

[10] Willcoxson L, Prosser M (1996). Kolb’s Learning Style Inventory (1985): review and further study of validity and reliability. Br J Educ Psychol.

[11] McCarthy B (1997). A Tale of Four Learners: 4MAT's Learning Styles. Educ Leadersh.

[12] Soloman BA, Felder RM Index of learning styles questionnaire. http://www.engr.ncsu.edu/learningstyles/ilsweb.

[13] Reid G (2005). Learning styles and inclusion. Sage;.

[14] Fleming N, Baume D (2006). Learning Styles Again: VARKing up the right tree! Edu Develop.

[15] Alfawzan A, Alfawzan O, Alessa R, Alturki A, Alshiha K, Omair A, Agha S, Mahzari MM (2020). An assessment of learning styles among undergraduate medical students at King Saud Bin Abdulaziz University (KSAUHS), King Saud University (KSU) and Imam Mohammad Ibn Saud Islamic University (IMSIU). Res Sq.

[16] Gustin MP, Abbiati M, Bonvin R, Gerbase MW, Baroffio A (2018). Integrated problem-based learning versus lectures: a path analysis modelling of the relationships between educational context and learning approaches. Med Educ Online.

[17] Wood SJ, Woywodt A, Pugh M, Sampson I, Madhavi P (2015). Twelve tips to revitalise problem-based learning. Med Teach.

[18] Hernández-Torrano D, Ali S, Chan CK (2017). First year medical students' learning style preferences and their correlation with performance in different subjects within the medical course. BMC Med Educ.

[19] Jiraporncharoen W, Angkurawaranon C, Chockjamsai M, Deesomchok A, Euathrongchit J (2015). Learning styles and academic achievement among undergraduate medical students in Thailand. J Educ Eval Health Prof.

[20] Shakeri F, Ghazanfarpour M, MalaKoti N, Soleimani Houni M, Rajabzadeh Z, Saadat S (2022). Learning Styles of Medical Students: A Systematic Review. Med Edu Bullet.

[21] Rezigalla AA, Ahmed OY (2019). Learning style preferences among medical students in the College of Medicine, University of Bisha, Saudi Arabia 2018. Adv Med Educ Pract.

[22] Penson J (2010). Development of future faculty teaching skills. Commun Agric Appl Biol Sci.

[23] Ramasamy DP, Pasupathy S (2021). Impact of Curriculum Change on Outcomes.

[24] Saffari M, Rshidi Jahan H, Mahmoudi N, Pakpour A, Sanaeinasab H (2016). Relationship of Learning Styles in Students of Health Sciences with Lifelong Learning. Iran J Health Educ Health Promot.

[25] Fahim A, Rehman S, Fayyaz F, Javed M, Alam MA, Rana S (2021). Identification of Preferred Learning Style of Medical and Dental Students Using VARK Questionnaire. Biomed Res Int.

[26] Mashhood S, Mashhood-uz-Zafar Farooq MF, Fahim MK (2018). Medical student’s preferred learning style. Pak J Surg.

[27] Koohestani HR, Baghcheghi N (2020). A comparison of learning styles of undergraduate health-care professional students at the beginning, middle, and end of the educational course over a 4-year study period (2015-2018). J Educ Health Promot.

[28] Aldosari MA, Aljabaa AH, Al-Sehaibany FS, Albarakati SF (2018). Learning style preferences of dental students at a single institution in Riyadh, Saudi Arabia, evaluated using the VARK questionnaire. Adv Med Educ Pract.

[29] Balasubramaniam G, Indhu K (2016). A study of learning style preferences among first year undergraduate medical students using VARK model. Edu Med J.

[30] Nuzhat A, Salem RO, Hamdan NA, Ashour N (2013). Gender differences in learning styles and academic performance of medical students in Saudi Arabia. Med Teach.

[31] ELsayed M, Mohsen D, Dogheim R, Zain H, Ahmed D (2016). Assessment of learning styles for medical students using VARK questionnaire. Int J Manage Appl Sci.

[32] Othman N, Amiruddin MH (2010). Different perspectives of learning styles from VARK model. Procedia Soc Behav Sci.

[33] Pellón M, Nome S, Arán A (2013). Relationship between learning styles and academic performance of fifth graders enrolled in the medical course. Rev Bras Oftalmol.

[34] Johnson S, Mathews C, Alawam KA, Saeed AA, Alshagag H, Qasem H (2022). Learning style preferences assessed by Kolb's learning style inventory among respiratory therapy students in Saudi Arabia. Ind J Resp Care.

[35] Ibrahim M, Al-Shahrani A (2018). Implementing of a problem-based learning strategy in a Saudi medical school: requisites and challenges. Int J Med Educ.

